# Immature Oocytes in “Apparent Empty Follicle Syndrome”: A Case Report

**DOI:** 10.1155/2010/367505

**Published:** 2010-03-30

**Authors:** Teraporn Vutyavanich, Waraporn Piromlertamorn, Jason Ellis

**Affiliations:** Division of Reproductive Medicine, Department of Obstetrics and Gynecology, Faculty of Medicine, Chiang Mai University, Chiang Mai 50200, Thailand

## Abstract

Empty follicle syndrome (EFS) is a condition in which no oocytes are obtained after an apparently successful ovarian stimulation. Genuine EFS (GEFS) is differentiated from false EFS by an optimal level of human chorionic gonadotropin on the day of oocyte retrieval. Some believe that GEFS does not exist and that it is only a reflection of the margin of error attendant upon the procedure of oocyte aspiration. Others believe that GEFS is caused by dysfunctional folliculogenesis, resulting in early atresia of oocytes. In this report, we present a case of apparent GEFS, in which immature oocytes were identified after filtration of follicular aspirates. Our findings suggest that delayed maturation of oocyte cumulus complexes in response to HCG might be an etiologic mechanism in some cases of GEFS. This creates a situation similar to the aspiration of immature follicles, where germinal vesicle-stage oocytes with dense scanty cumulus cells are often difficult to identify under a dissecting microscope.

## 1. Introduction

Empty follicle syndrome (EFS) is a condition in which no oocytes are retrieved after an apparently successful ovarian stimulation [1, 2]. Its incidence has been estimated to be around 0.2%–7% [1, 3–6]. This situation is frustrating and highly stressful to both patients and the IVF team.

Empty follicle syndrome (EFS) was first described by Coulam et al. [7] in 4 patients with unexplained infertility, who underwent 5 cycles of in vitro fertilization. They suggested that EFS might represent a so far unknown cause of infertility. In subsequent studies, EFS was reported in couples with practically any causes of infertility [1, 2]. For simplicity, Stevenson and Lashen [1] classified EFS into genuine and false EFS. False EFS was defined as a failure to retrieve oocytes in the presence of low *β*-HCG level. Possible etiologies include inappropriate administration of HCG [5, 8], defects in the biological preparations of HCG [6], or individual variation in the bioavailability and metabolism of HCG [2]. Genuine EFS was defined as unsuccessful oocyte retrieval after apparently normal follicular development with optimal *β*-HCG levels on the day of oocyte retrieval [1]. Some believed that genuine EFS was due solely to technical difficulties during oocyte aspiration [9, 10]. Given the oocyte aspiration retrieval rate of only 80%, despite the use of follicular flushing, van Heusden et al. [9] calculated the chance of failure to recover at least one oocyte in their 14 EFS cases to be 0.064%–4%. This number was coincidentally close to the reported prevalence of EFS in general and was used as one argument against the existence of EFS as a clinical entity. Ben-Shlomo et al. [4] reviewed 26 patients with EFS and found no differences between successful and unsuccessful cycles with respect to stimulation protocols, hormonal response pattern or the number of large follicles (≥15 mm) detected by sonography. Ten of their patients had been pregnant in the past and twenty had at least one other IVF cycle that yielded oocytes. The authors, therefore, concluded that failure to retrieve oocytes should be regarded as a “sporadic event” rather than a syndrome. They also noted that 4 of these patients had a history of poor response to ovarian stimulation, implying that perhaps ovarian dysfunction could play a role. Others [6, 11] also suggested dysfunctional folliculogenesis, a clinical dysfunction in which abnormal granulosa cell function leads to altered oocyte growth and early atresia, as the likely cause of GEFS. Zreik et al. [12] found that patients with one GEFS cycle had a 20% risk of recurrence in later IVF cycles. The risk of recurrence increased with age. Those between 35–39 years of age had a recurrence risk of 24%, increasing to 57% for those >40 years old. They suggested ovarian aging, possibly through altered granulosa cell function and apoptosis, might be involved in the etiology of EFS, and especially in its recurrence. Onalan et al. [13] described recurrent GEFS in 3 stimulated cycles in 2 sisters with congenital hearing loss and postulated an inherited genetic factor as its etiology. However, further reported cases and more detailed molecular investigations are necessary to verify this claim.

In this report, we present a case of apparent genuine EFS. The interesting finding was that the follicular aspirates, which contained no oocytes by conventional observation, were found to harbor immature (germinal vesicle) oocytes upon filtration through a cell strainer.

## 2. Case Report

A 32-year-old Thai woman was referred to us for in vitro fertilization (IVF) treatment in June, 2008. Previous history revealed that she had been treated for her unexplained infertility at a provincial hospital in 2005. She was pregnant after timing of sexual intercourse on her third cycle of clomiphene citrate. Unfortunately, the pregnancy ended in a complete spontaneous miscarriage at a gestational age of 5 weeks and 3 days. After the abortion, she tried to conceive on her own without success. She went back to her previous doctor, who treated her with 3 cycles of clomiphene, with timing of sexual intercourse, followed by 2 cycles of superovulation and intrauterine insemination (IUI). After unsuccessful treatments, the patient had diagnostic laparoscopy performed in 2006. Minimal pelvic endometriosis was found and visible implants were cauterized. Dye injection revealed patency of both fallopian tubes. She was treated with leuprorelin acetate 3.75 mg (Enantone; Takeda, Osaka, Japan) monthly for 6 months, followed by another 2 cycles of superovulation and IUI, to no avail.

A long luteal protocol was used for her IVF. She was given 1.88 mg of leuprorelin acetate (Enantone) on June 30th, 2008. Blood was taken to confirm down—regulation on July 9th, 2008 (cycle day 3), and recombinant follicle stimulating hormone (rFSH—Puregon; Organon, Oss, the Netherlands) 150 IU per day was started. After three doses of rFSH 150 IU daily, blood was again taken and revealed an estradiol level of 86.9 pg/mL. The dose of rFSH was increased to 200 IU per day for another two days and repeated blood test revealed satisfactory rise of estradiol level. Transvaginal ultrasound was performed 8 days later, revealing 12 follicles as follows: 2 × 15 mm, 1 × 14 mm, 8 × 13 mm and 1 × 12 mm follicles. Ultrasound was repeated 2 days later, revealing 2 × 19, 1 × 18, 4 × 17, 4 × 15 and 1 × 13 mm follicles. Human chorionic gonadotropin (HCG; Pregnyl, Organon) 10,000 IU was administered intramuscularly and oocyte pick-up was performed 36 hours later. At oocyte retrieval, there was minimal fluid in the cul-de-sac. All 12 follicles were aspirated using a 16G double-lumen aspiration needle (catalog no. K-OPSD-1635-A-L; Cook Medical, Brisbane, Australia). Flushing was performed twice after each follicular aspiration, using heparinized Dublecco's phosphate-buffered saline (DPBS; Gibco, BRL, NY, USA). Follicular and flushing fluid from consecutive follicles were aspirated collectively into warm 50-mL culture flasks (Nunc, Roskilde, Denmark). In our setting, two embryologists worked side by side, routinely checking follicular aspirates previously examined by the other. As no oocyte was obtained, we examined the aspirates a third time, before a tentative diagnosis of “empty follicle syndrome” was made.

The patient confirmed that she had HCG injected by a qualified nurse at the correct time, approximately 36 hours before oocyte retrieval. Blood was immediately taken for hormonal study. Her *β*-HCG level was found to be 851 IU/L, FSH 12 IU/L, LH < 0.1 IU/L, estradiol 2,637 pg/mL and progesterone 37.5 ng/mL. Out of frustration, we decided to have a closer look at the aspirates by filtering them through a 70-*μ*m cell strainer (BD Falcon, Bedford, MA, USA). Unfortunately, half of the follicular aspirates had already been discarded by this time. To our great surprise, 4 immature (germinal vesicle-stage) oocytes were identified in the remaining fluid. Two oocytes had a very scant amount of tightly-packed cumulus cells and the other two were nearly cumulus-free, making it difficult or impossible to identify them without the aid of a cell strainer. As the oocytes had been left standing at room temperature for too long (>3 hours), we decided to discard them. The patient was counseled regarding EFS. She was advised that the chance of a recurrence in her case was negligible, and we would do our best in her next cycle.

The patient came back for a second IVF attempt 4 months after the incident. Human menopausal gonadotrophin (HMG; Menogon; Ferring GmbH, Kiel, Germany) 225 IU per day was started on cycle day 3 for 9 consecutive days. Ganirelix 0.25 mg (Orgalutran; Organon) was given daily on cycle days 8–11. HCG 10,000 IU (Pregnyl, Organon) was given on cycle day 12 and oocyte retrieval was done 36 hours later (on cycle day 14). Twelve oocytes were obtained and 9 embryos were produced by IVF. Two embryos were transferred and 7 embryos were cryopreserved by ultra-rapid freezing technique. Urine pregnancy test was positive two weeks after the embryo transfer. Transvaginal ultrasound examination was performed seven weeks after the embryo transfer. A single gestational sac, with yolk sac and fetal heart activity, was visualized inside the uterine cavity. At the time of this report, the pregnancy was still ongoing.

## 3. Discussion

Premature LH surge was an unlikely explanation for GEFS in our case as a single half dose of leuprolide depot was used in a long down-regulation protocol to prevent its occurrence. In a recent randomized study, Isikoglu et al. [14] showed that a single half-dose of leuprolide depot was as effective as the multi-dose protocol for pituitary desensitization. In their study [14], premature LH surge did not occur in any of the 51 cases who received this medication. The presence of intact follicles at the time of oocyte retrieval also provided further evidence against the occurrence of premature LH surge in our case.

The usual retrieval rate of oocyte aspiration is 80% [9]. The mathematical chance for failure to recover any oocyte in our case, with 12 follicles, would be only 4 out of 10 million. Such an extreme probability suggested that the margin of error inherent in the procedure of oocyte aspiration itself would be an unlikely explanation for EFS in our reported case.

Initially, the patient responded poorly to ovarian stimulation, making it necessary to increase the doses of rFSH. It was possible that abnormal folliculogenesis could have occurred at the beginning of the cycle, as in other reported cases of GEFS [4, 6]. Normal levels of estradiol and progesterone on the day of oocyte retrieval, however, did not support the contention by some authors [6, 11] that GEFS, at least in our case, was due to early atresia of the oocytes. Abnormal folliculogeneis could possibly modify the follicular response to an ovulatory dose of HCG. Interestingly, Meniru and Craft [15] suggested that there could be a delayed detachment of oocyte-cumulus complex (OCC) from the follicular wall following HCG injection. In their report, the first dose of HCG raised the plasma level of progesterone, but no oocytes were retrieved from the aspiration of one ovary. They repeated HCG injection and were able to obtain 20 oocytes from the other ovary at a second retrieval 24 hours later. Hassan et al. [16] also reported similar findings, suggesting that some patients might need a longer exposure time to HCG in order for their OCCs to detach from the follicle wall. In a similar way to our case, HCG could induce normal progesterone production, but the final maturation of the OCCs was somehow delayed. As immature oocytes were not free-floating in the follicular fluid at the time of oocyte retrieval, the yield of follicular aspiration, even with repeated flushing of the follicles, would be markedly reduced. We postulated that the presence of fewer OCCs in the follicular aspirates, together with the unique features of immature OCCs, made it difficult for the average embryologist to identify them. Given the normal level of progesterone, we questioned the role of a second HCG injection as suggested by some authors [15, 16]. Perhaps oocyte maturation in response to the first dose of HCG just required more time than 36 hours and mature oocytes could be aspirated later on, regardless of the second HCG injection.

Recently, our center began to explore the use of in vitro maturation (IVM) of oocytes from unstimulated cycles in patients with polycystic ovary syndrome (PCOS). The follicular aspirates from IVM cases were more heavily contaminated with blood but they were, to a certain extent, similar to that seen in this patient. Although we found only 4 GV oocytes, it was possible that more immature oocytes might have been missed in the remaining aspirate that was discarded. Our PubMed search from 1986 to 2009, using the keywords “empty follicle syndrome” or “failed oocyte retrieval,” revealed one case report [17] that also found 1 zona-free germinal vesicle-stage oocyte in the follicular aspirate of 1 GEFS case.

In conclusion, some cases of genuine EFS might be caused by a delayed maturation of OCCs in response to HCG. The situation was similar to oocyte retrieval from immature follicles. The lower number of immature oocytes in the aspirates and the difficulty in identifying them could lead to a mistaken diagnosis of GEFS.
